# Cancer-testis antigen SLLP1 represents a promising target for the immunotherapy of multiple myeloma

**DOI:** 10.1186/s12967-015-0562-5

**Published:** 2015-06-20

**Authors:** Sara Yousef, Johanna Heise, Nesrine Lajmi, Katrin Bartels, Nicolaus Kröger, Tim Luetkens, Djordje Atanackovic

**Affiliations:** Hematology and Hematologic Malignancies, Huntsman Cancer Institute, University of Utah, Salt Lake City, UT USA; Stem Cell Transplantation, University Medical Center Hamburg-Eppendorf, Hamburg, Germany; Oncology/Hematology/Bone Marrow Transplantation with the Section Pneumology, University Medical Center Hamburg-Eppendorf, Hamburg, Germany; Multiple Myeloma Program and Cancer Immunotherapy, Division of Hematology and Hematologic Malignancies, University of Utah/Huntsman Cancer Institute, Room HCI 4265, 2000 Circle of Hope Drive, Salt Lake City, UT 84112 USA

**Keywords:** Cancer-testis antigens, SLLP1, Multiple myeloma, Tumor immunology, Immunotherapy, Adoptive cell transfer

## Abstract

**Background:**

Most patients with multiple myeloma (MM) will relapse after an initial response and eventually succumb to their disease. This is due to the persistence of chemotherapy-resistant tumor cells in the patients’ bone marrow (BM) and immunotherapeutic approaches could contribute to eradicating these remaining cells. We evaluated SLLP1 as a potential immunotherapeutic target for MM.

**Methods:**

We determined SLLP1 expression in myeloma cell lines and 394 BM samples from myeloma patients (n = 177) and BM samples from healthy donors (n = 11). 896 blood samples and 64 BM samples from myeloma patients (n = 263) and blood from healthy donors (n = 112) were analyzed for anti-SLLP1 antibodies. Seropositive patients were evaluated regarding SLLP1-specific T cells.

**Results:**

Most cell lines showed SLLP1 RNA and protein expression while it was absent from normal BM. Of 177 patients 41% evidenced SLLP1 expression at least once during the course of their disease and 44% of newly diagnosed patients were SLLP1-positive. Expression of SLLP1 was associated with adverse cytogenetics and with negative prognostic factors including the patient’s age, number of BM-infiltrating plasma cells, serum albumin, β_2_-microglobulin, creatinine, and hemoglobin. Among patients treated with allogeneic stem cell transplantation those with SLLP1 expression showed a trend towards a reduced overall survival. Spontaneous anti-SLLP humoral immunity was detectable in 9.5% of patients but none of the seropositive patients evidenced SLLP1-specific T cells. However, antigen-specific T cells could readily be induced in vitro after stimulation with SLLP1.

**Conclusions:**

SLLP1 represents a promising target for the immunotherapy of MM, in particular for the adoptive transfer of T cell receptor-transduced T cells.

## Background

Multiple myeloma (MM) is an incurable plasma cell (PC) malignancy, which develops in the bone marrow (BM) and eventually causes renal insufficiency, immunosuppression with repeated infections, anemia, and bone lesions with hypercalcemia. There have been significant therapeutic advances over the past decade and the median survival of myeloma patients has increased to approximately 6 years [1]. However, most patients will still eventually suffer a fatal relapse after an initially effective therapy. This is due to the persistence of chemotherapy-resistant myeloma-propagating cells [2–4] in the BM even after destruction of the bulk of tumor cells by conventional therapies [5–8] and, accordingly, the disease will become more and more refractory to chemotherapy after each additional line of treatment. We, therefore, believe that MM needs to be attacked from different biological angles by a variety of modalities, including immunotherapeutic approaches, to destroy the tumor bulk as well as myeloma-propagating residual disease and eventually achieve cures.

One prerequisite for the development of effective cancer immunotherapies is the identification of appropriate target antigens. Cancer-testis antigens (CTA) are a family of proteins which show an expression restricted to different malignancies and normal germ-line tissues and are capable of eliciting spontaneous cellular and humoral immune responses against a variety of tumors [9]. Antigen SLLP1, which is encoded by gene SPACA3, was first described as a unique, non-bacteriolytic lysozyme-like protein expressed in the acrosome of human sperm [10]. Later, SLLP1 was found to be a member of the CTA family of antigens and a preliminary study detected its expression in different hematologic malignancies [11]. However, so far no broad analysis has been performed regarding SLLP1 expression in MM, its expression has never been correlated with clinicopathological characteristics of the patients, and its ability to evoke specific humoral and T cell responses has not been explored.

## Methods

### Cell lines

Human myeloma cell lines AMO-1, MOLP-8, RPMI-8226, KMS-12-BM, EJM, IM-9, U-266, OPM-2, and LP-1, chronic myeloid leukemia cell line K562, and human embryonic kidney cell line 293 were newly obtained from the German Collection of Microorganisms and Cell Cultures (DSMZ, Braunschweig, Germany). Two additional human myeloma cell lines, Brown and SK-007, were provided by the New York branch of the Ludwig Institute for Cancer Research (LICR). Upon arrival in our laboratory, the authenticity of the cell lines was verified using cytology and flow cytometry. All cell lines were maintained in RPMI 1640 medium (Invitrogen, Carlsbad, CA, USA) with penicillin–streptomycin (Invitrogen) and 10% fetal calf serum (Lonza, Basel, Switzerland).

### Patients and healthy donors

For SLLP1 expression analyses we consecutively collected 394 bone marrow samples from a total of 177 myeloma patients. In addition, BM samples of 11 different healthy donors, who provided BM for allogeneic stem cell transplantation, were obtained. For the analyses of antibody responses a total of 896 blood samples and 64 BM samples were obtained from a different cohort of 263 myeloma patients, and 112 blood samples were collected from healthy blood donors. BM and blood samples from patients were obtained during routine diagnostic procedures. Healthy subjects and patients, who were admitted for treatment at the University Medical Center Hamburg-Eppendorf, gave written informed consent in accordance with the revised version of the Declaration of Helsinki. The study protocol had been approved by the local ethics committee (decision number OB-038/06). For the preparation of plasma samples, whole BM or peripheral blood was centrifuged at 680*g* and the supernatants were frozen at −80°C. Mononuclear cells were isolated from blood and BM samples by density gradient centrifugation.

### Reverse transcription-polymerase chain reaction (RT-PCR)

Total RNA was extracted from BM mononuclear cells (BMMC) and myeloma cell lines using the RNeasy Mini kit (Qiagen, Hilden, Germany) and reverse transcribed to complementary DNA (cDNA) applying avian myeloblastosis virus (AMV) reverse transcriptase (Promega, Madison, WI, USA). RNA derived from human testis was obtained from Applied Biosystems (Carlsbad, CA, USA). Primers for qualitative PCR amplification of SLLP1 cDNA (Forward: 5′-AAGCTCTACGGTCGTTGTGAACTG-3′; Reverse: 5′-CTAGAAGTCACAGCCATCCACCCA-3′) and the cDNA for the housekeeping gene glyceraldehyde-3-phosphate dehydrogenase (GAPDH; Forward: 5′-TGATGACATCAAGAAGGTGG-3′; Reverse: 5′-TTTCTTACTCCTTGGAGGCC-3′) were obtained from MWG Biotech (Ebersberg, Germany). Conventional PCR was performed as previously described [12]. All RT–PCR experiments were performed at least twice. To assess primer specificity, PCR products were analyzed repeatedly by DNA sequence analysis.

### Western blot analysis

Whole cell protein extracts were prepared in RIPA buffer containing a cocktail of protease inhibitors (Sigma, Steinheim, Germany). Testis lysate used as a positive control was obtained from Abnova (Taipei, Taiwan). 293 cells were transfected with an SLLP1 expression plasmid (Origene, Rockville, MD) using Lipofectamine 2000 (Lifetechnologies) and harvested after 3 days. Protein concentrations were determined by BCA assay (Thermo Scientific) and immunoblot analysis was performed as previously described [13] applying 80 µg of protein per lane. The primary antibodies were a rabbit polyclonal antibody against human SLLP1 (Sigma) used at a dilution of 1:1,000 and a mouse anti-human monoclonal antibody against β-actin (ACTB; Cell Signaling Technology, Danvers, MA) used at a dilution of 1:3,000. Secondary antibodies were an HRP-labeled anti-rabbit monoclonal antibody (R&D Systems, Minneapolis, MN, USA) used at a dilution of 1:2,000 or an HRP-labeled anti-mouse monoclonal antibody (R&D Systems, Minneapolis, MN, USA) used at a dilution of 1:3,000, respectively. Specific antibody binding was visualized by chemiluminescence (PerkinElmer, Waltham, MA, USA).

### Flow cytometry

For the analysis of cytoplasmic SLLP1 protein expression, myeloma cell lines were fixed using FACS Lysing Solution, followed by permeabilization with Permeabilizing Solution (both from BD Biosciences). Cells were stained with a rabbit polyclonal antibody against human SLLP1 (Sigma) or an appropriate isotype control antibody followed by incubation with a secondary FITC-conjugated goat anti-rabbit IgG antibody from Jackson ImmunoResearch (Suffolk, UK). Samples were analyzed using a FACSCalibur cytometer (BD Biosciences, Franklin Lakes, NJ, USA) and FlowJo software (Tree Star, Ashland, OR, USA).

### Enzyme-linked immunosorbent assay (ELISA)

A set of 20-mer SLLP1 peptides (n = 21) overlapping by 10 amino acids and spanning the complete protein sequence was obtained from Peptides&Elephants (Potsdam, Germany). Recombinant influenza nucleoprotein (NP) expressed in *E. coli* was purchased from Imgenex (San Diego, CA, USA), tetanus toxoid (TT) was provided by Chiron Behring (Marburg, Germany), and recombinant SSX-2 protein was provided by the LICR. 96-well-plates were coated over night at 4°C with recombinant protein or peptides diluted in PBS at a final concentration of 1 μg/ml. Plates were blocked with PBS containing 3% milk powder for 2 h at room temperature (RT). Sera were added to the plates and incubated for 2 h at RT. A secondary AP-conjugated anti-human-IgG antibody (Southern Biotech, Birmingham, AL, USA) was applied for 1 h at RT. Detection reagent para-nitrophenyl phosphate (PNPP; Southern Biotech) was added to the plates for 30 min and specific absorption was measured at 405 nm using a Sunrise™ ELISA reader (Tecan, Crailsheim, Germany).

### Generation of peptide-loaded dendritic cells

For the generation of dendritic cells (DC) monocytes were obtained through plastic adherence. Briefly, PBMCs were seeded into 75 cm^2^ flasks for 2 h. Non-adherent cells were removed by extensive washing. The remaining monocytes were incubated in DC medium CellGro (CellGenix, Freiburg, Germany) for 5 days in the presence of 200 IU/ml interleukin (IL)-4 (R&D Systems) and 100 IU/ml granulocyte macrophage colony-stimulating factor (GM-CSF; R&D Systems) to induce an immature DC phenotype. Restimulation with cytokines was performed on days 2 and 5. At day 6 a pool of peptides containing overlapping 20-mer SLLP1 peptides (Peptides&Elephants) at a concentration of 20 µM, respectively, was added to the culture. After 2 h a maturation cocktail was added containing tumor necrosis factor (TNF)-α (5 ng/ml), IL-1β (5 ng/ml), IL-6 (150 ng/ml; all R&D Systems), and prostaglandin (PGE)_2_ (1 µg/ml; Sigma). The maturation status of the DCs was assessed the next day by flow cytometry using anti-HLA DR, CD80, CD86, CD83, and CD14 monoclonal antibodies (BioLegend, San Diego, CA, USA). Peptide-loaded, mature DCs were either used directly or cryopreserved for later use.

### Induction of SLLP1-specific T cells

The monocyte-depleted PBMC fraction was seeded at ratio of 1:10 with autologous peptide-loaded DCs in 96-well plates in RPMI (5% human serum, 1% pen/strep) in the presence of IL-2 (Roche Diagnostics, Rotkreuz, Switzerland) and IL-7 (R&D Systems) at 10 ng/ml. Restimulation with cytokines and DCs was carried out weekly at ratios of 1:20 to 1:40. Specificity assessment was performed after 2–3 rounds of restimulation by interferon (IFN)-γ enzyme-linked immunosorbent spot (ELISPOT) assay as previously described [14]. Briefly, 2.5 × 10^4^ T cells were cocultured with 5 × 10^4^ autologous PHA blasts serving as T-APC [15] in the presence or absence of SLLP1 peptides overnight in anti-IFN-γ-coated (Mabtech, Stockholm, Sweden) nitrocellulose plates (Millipore, Bedford, MA, USA). Following development, spots were counted using an AID EliSpot reader and EliSpot software version 3.2.3 (Autoimmun Diagnostika, Strassberg, Germany). For enrichment and single cell isolation of SLLP1-specific T cells up to 1 × 10^6^ T cells were incubated with CFSE-labeled T-APC at a ratio of 1:1 in the presence or absence of cognate SLLP1 peptide in X-Vivo15 medium (Bio Whittaker, Verviers, Belgium). IFN-γ-positive T cells were FACS-sorted using the IFN-γ Secretion Assay (Miltenyi Biotech, Bergisch Gladbach, Germany) as described previously [16]. Clonality was determined by flow cytometry by the use of a set of 24 different TCR v beta antibodies (IO Test; Beckman Coulter). The TCR chain composition was assessed by PCR using a primer set against TRBV chains and TRAV chains as previously described [17, 18].

### Statistical analysis

Correlations between clinicopathological parameters and CT antigen expression were assessed using Pearson’s Chi-square test. Log-Rank test and Cox-regression analysis were performed for evaluation of survival and relapse in MM patients. Results were considered significant if p < 0.05.

## Results

### SLLP1 is expressed in myeloma cells but is absent from healthy bone marrow

**Figure 1:**
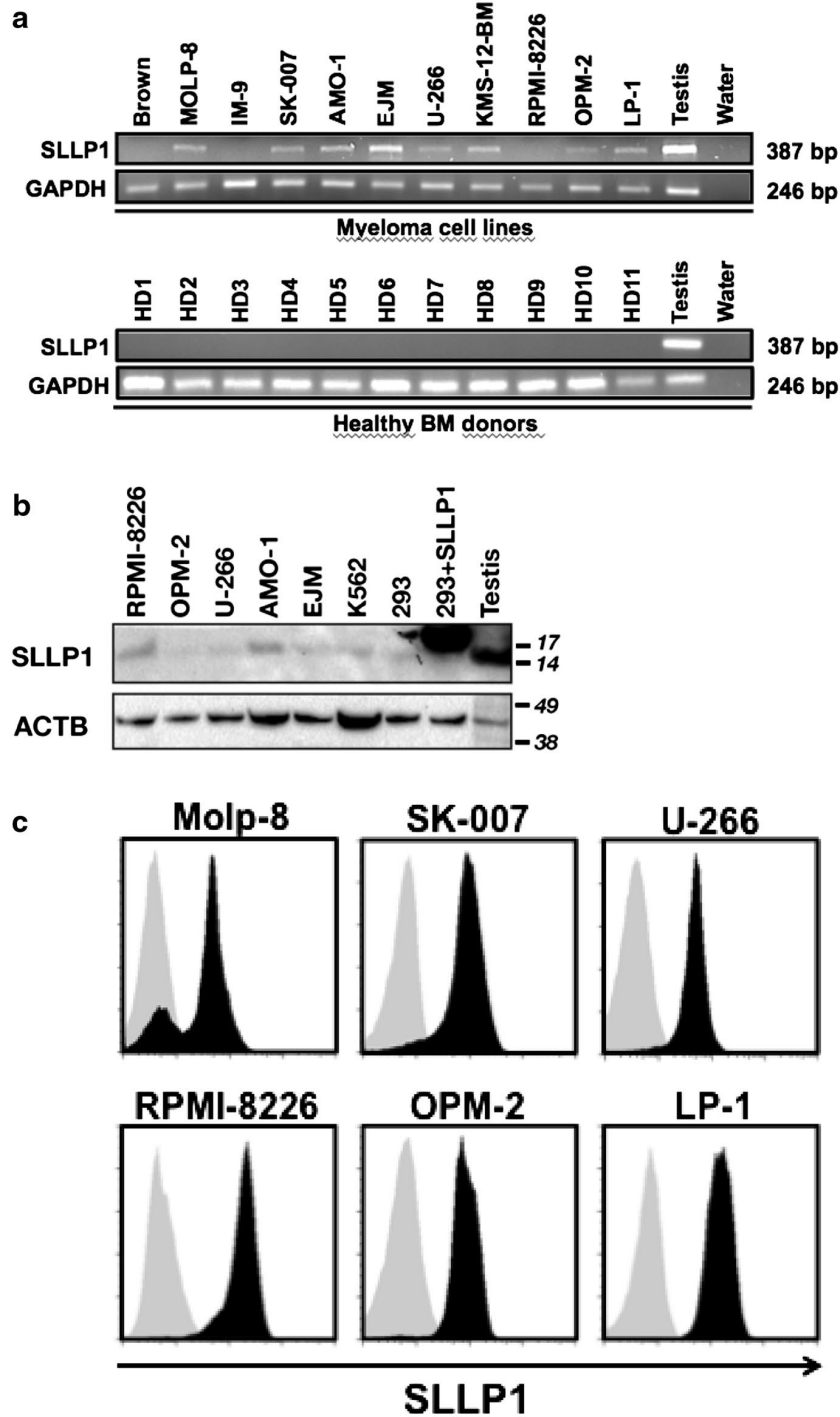
SLLP1 is specifically expressed by myeloma cells. **a** RNA expression of SLLP1 was analyzed by RT-PCR in 11 different myeloma cell lines (*upper row*) and bone marrow samples from 11 healthy donors (*lower row*). Human testis was used as a positive control and housekeeping gene GAPDH served as an internal loading control. **b** Expression of SLLP1 in 5 myeloma cell lines, chronic myeloid leukemia cell line K562, 293 cells, 293 cells transfected with an SLLP1 expression plasmid and human testis lysate was confirmed by western blot analysis. Beta actin (ACTB) was used as an internal control. **c** Myeloma cell lines (n = 6) were analyzed for SLLP1 expression on a single-cell level applying cytoplasmic staining followed by flow cytometry. *Grey areas* indicate staining intensity with an irrelevant isotype control, *black areas* show staining with anti-SLLP1 antibody.

In a first step, we analyzed SLLP1 RNA expression in different myeloma cell lines using qualitative RT-PCR. We found the majority (8/11) of cell lines tested to evidence SLLP1 expression (Figure 1a). As expected for a cancer-testis antigen, SLLP1 RNA was also present in normal testis tissue but, importantly, it was absent from the whole BM of 11 healthy donors analyzed (Figure 1a) thus indicating a tumor-restricted expression of SLLP1. Next, we confirmed SLLP1 protein expression in myeloma cell lines, testis lysate, and 293 cells transfected with an SLLP1 expression plasmid using western blot analyses (Figure 1b). The observed band at 15 kDa corresponds to the calculated and previously described molecular weight of cleaved SLLP1 [11]. Applying flow cytometry for the analysis of SLLP1 on a single-cell level we did not detect convincing expression of SLLP1 on the surface of any of our myeloma cell lines (data not shown). However, we observed strong SLLP1 protein expression in the cytoplasm of all lines that had also evidenced presence of the CTA as indicated by western blot (Figure 1c).

### SLLP1 expression in myeloma patients and its relation to clinicopathological characteristics and prognosis

Analyzing the clinicopathological characteristics of all 177 patients (Table 1), we found the typical patient to be male with a median age of 54 years (range 28–83 years). Importantly, there were no significant differences with regard to age and sex between the SLLP1-positive and the SLLP-negative subgroups of patients. Most patients were diagnosed at later stages of the disease and the most common idiotype was IgG kappa. While 52 (29%) of the patients were included immediately after initial diagnosis, most patients had already received therapy before this study was initiated. Out of all 177 patients approximately 41% showed expression of SLLP1 RNA in their BM at least once throughout the whole course of their disease (Table 1). Looking at only newly diagnosed patients we found about 44% to be SLLP1-positive (data not shown). The only clinicopathological parameter correlating with the expression of SLLP1 were cytogenetics with 9 out of 11 patients with a t(4;14) translocation being SLLP1-positive (Table 1). This finding also held true when we focused on the untreated patients with all 6 patients evidencing a t(4;14) being SLLP1-positive (p < 0.004).

**Table 1:** Patient characteristics and association with SLLP1 expression

Characteristics	SLLP1-positive	SLLP1-negative	Sig.
Total (N = 177)	72 (40.7)	105 (59.3)	
Age (years)			
≤60	45 (36.6)	78 (63.4)	0.094
>60	27 (50.0)	27 (50.0)	
Sex			
Male	46 (41.1)	66 (58.9)	0.889
Female	26 (40.0)	39 (60.0)	
Initial stage			
I	10 (38.5)	16 (61.5)	0.928
II	10 (38.5)	16 (61.5)	
III	52 (41.6)	73 (58.4)	
Heavy chain isotype			
IgG	41 (40.2)	61 (59.8)	0.246
IgA	22 (47.8)	24 (52.2)	
IgM	1 (100.0)	0 (0.0)	
Light chain	8 (28.6)	20 (71.4)	
Light chain isotype			
Kappa	43 (37.7)	71 (62.3)	0.281
Lambda	29 (46.0)	34 (54.0)	
Cytogenetics			
Normal karyotype	25 (43.1)	33 (56.9)	0.345
Del13q14	31 (58.5)	22 (41.5)	0.069
Del17p13	9 (64.3)	5 (35.7)	0.192
t(4;14)	9 (81.8)	2 (18.2)	*0.018*
Other	13 (38.2)	2 (61.8)	0.198

A total of 177 patients with MM were classified according to the clinical features of their disease. Information on cytogenetics (N = 136) was available for fewer patients. A patient was considered SLLP1-positive if at least one of all samples collected per patient evidenced SLLP1 RNA expression. Importantly, median follow-up time was not significantly different between SLLP1-postive and -negative patients (data not shown). P values as calculated by Chi-square test are indicated in the column on the far right.

From our MM patients we were able to collect a total of 394 BM samples with a median number of two (range 1–9) samples per patient over a median period of 3.8 months (range 0–53 months). Of all these samples about one quarter evidenced expression of SLLP1 RNA (Table 2). Correlating the expression data with the clinical data of the patients at the time the BM sample was collected, we found SLLP1 expression to be associated with a number of negative prognostic factors including the patient’s age, the number of BM-infiltrating plasma cells, as well as serum levels of albumin, β_2_-microglobulin, hemoglobin, and creatinine. Importantly, we also observed a strong correlation with the treatment status of the patient with untreated patients most frequently showing SLLP1 expression in their BM and patients who had undergone alloSCT evidencing the lowest positivity rates (Table 2).

**Table 2:** Sample characteristics and association with SLLP1 expression

Characteristics	SLLP1-positive	SLLP1-negative	Sig.
Total (N = 394)	99 (25.1)	296 (74.9)	
Age at time of sample collection (years)			
≤60	64 (21.1)	240 (78.9)	*0.001*
>60	35 (38.9)	55 (61.1)	
BM-infiltrating plasma cells (%)			
<10	24 (11.6)	183 (88.4)	*0.000*
10–30	19 (33.3)	38 (66.7)	
≥30	37 (53.6)	32 (46.4)	
Serum paraprotein (g/dL)			
<5 (IgG), <3 (IgA)	8 (14.3)	48 (85.7)	0.061
5–7 (IgG), 3–5 (IgA)	7 (24.1)	22 (75.9)	
>7 (IgG), >5 (IgA)	57 (30.0)	133 (70.0)	
Involved serum free light chains (mg/L)			
≤100	6 (30.0)	14 (70.0)	0.129
>100	3 (10.0)	27 (90.0)	
Serum albumin (g/dL)			
≥3.5	67 (21.8)	241 (78.2)	*0.006*
<3.5	19 (43.2)	25 (56.8)	
β_2_-microglobulin (mg/L)			
<3.5	22 (18.6)	96 (81.4)	*0.019*
≥3.5	18 (35.3)	33 (64.7)	
Serum LDH (U/L)			
≤300	75 (23.0)	251 (77.0)	0.082
>300	13 (35.1)	23 (63.9)	
Hemoglobin (g/dL)			
>10.0	6 (27.3)	16 (72.7)	*0.043*
9.9–8.5	16 (41.0)	23 (59.0)	
<8.5	55 (22.4)	190 (77.6)	
Serum calcium (mmol/L)			
≤2.6	79 (22.8)	267 (77.2)	*0.015*
>2.6	9 (47.4)	10 (52.6)	
Serum creatinine (mg/dL)			
≤2.0	78 (22.5)	269 (77.5)	*0.008*
>2.0	9 (50.0)	9 (50.0)	
Treatment status			
Untreated	25 (44.6)	31 (55.4)	*0.003*
Chemotherapy	8 (22.2)	28 (77.8)	
AutoSCT	19 (29.2)	46 (70.8)	
AlloSCT	47 (19.9)	189 (80.1)	

A total of 394 samples from 177 patients with MM were classified according to clinical features at the time the given sample was collected. Information on percentage of BM-infiltrating plasma cells (N = 333), serum paraprotein (N = 275), albumin (N = 352), β_2_-microglobulin (N = 169), LDH (N = 362), hemoglobin (N = 306), calcium (N = 365), and creatinine (N = 365) were available for fewer samples. Free light chains are only given for the 50 samples from patients with light chain myeloma. P values as calculated by Chi-square test are indicated in the column on the right.

*BM* bone marrow, *LDH* lactate dehydrogenase.

**Figure 2:**
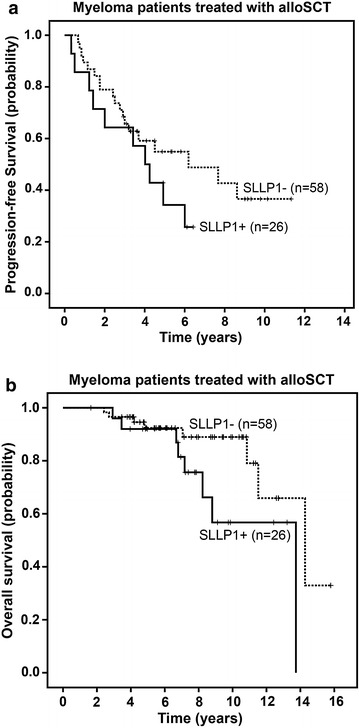
Expression of SLLP1 correlates with a worse prognosis in patients treated with alloSCT. A total of 84 patients who had received alloSCT were divided into to two groups according to the SLLP1 expression status in their bone marrow as determined by RT-PCR. Curves represent Kaplan–Meier estimates of progression-free survival (**a**) and overall survival (**b**) from application of alloSCT until end of observation.

Patients who had received alloSCT also seemed to represent a unique subgroup when it came to the relation between SLLP1 expression in the BM and the patients’ prognosis. We did not observe an effect of BM-standing SLLP1 expression on the clinical outcome of all 177 myeloma patients included or of the 52 newly diagnosed and untreated patients (data not shown). However, we found that among patients treated with alloSCT those who had evidenced SLLP1 expression in their BM at least at one time point after transplant showed a progression-free survival (Figure 2a), which was not significantly shorter (p = 0.21), and a trend (p = 0.07) towards a shorter overall survival (Figure 2b) compared to patients who always remained SLLP1-negative.

### Myeloma patients develop spontaneous antibody responses against SLLP1

**Figure 3:**
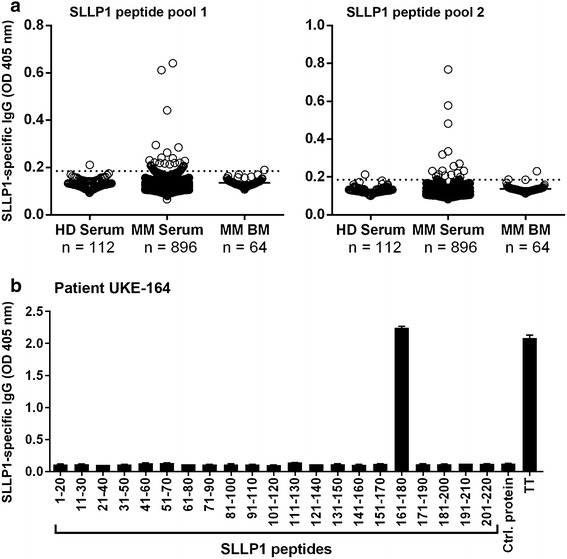
Myeloma patients occasionally develop spontaneous IgG antibody responses against SLLP1. Based on the unavailability of recombinant full-length SLLP1 protein with a sufficient quality, we decided to use SLLP1 20-mer peptides overlapping by 10 amino acids for the detection of spontaneous anti-SLLP1 IgG antibody responses. **a** For the ELISA assays, plates were coated with two pools of peptides containing SLLP1 peptides 1–10 (pool SLLP1-1; *left*) and 11–21 (pool SLLP1-2; *right*), respectively, *Dots* indicate intensity of the signal as measured by an absorbance reader. **b** Target epitopes of the SLLP1-specific antibody response of MM patient UKE-164 were identified using single overlapping 20-mer peptides spanning the complete sequence of SLLP1 in an ELISA assay. Recombinant influenza nucleoprotein (NP) and tetanus toxoid (TT) were used as positive controls, recombinant SSX-2 protein served as an irrelevant control protein. *Bars* indicate intensity (mean + SEM) of the signal against the given epitope. All results were validated by three independent experiments.

We next asked the question whether SLLP1 would be capable of evoking spontaneous humoral immune responses in patients with MM. Using two pools of overlapping SLLP1 peptides in an ELISA assay we analyzed a total of 896 peripheral blood and 64 BM plasma samples from 263 myeloma patients. We found SLLP1-specific IgG antibodies in a total of 44 (4.9%) serum samples. 22 (2.5%) samples were positive for peptide pool SLLP1–1, 15 (1.7%) for peptide pool SLLP1–2, and 7 (0.8%) sera evidenced antibodies against both peptide pools (Figure 3a). We also found anti-SLLP1 antibody responses in 1 (1.6%) and 3 (4.7%) of BM-derived sera from 64 myeloma patients for peptide pools SLLP1–1 and SLLP1–2, respectively (Figure 3a). Overall, out of all patients, 25 (9.5%) evidenced anti-SLLP1 antibody responses at least once throughout the course of their disease.

For some seropositive patients we were even able to define immunodominant linear epitopes within the SLLP1 protein. In the case of myeloma patient UKE-164, for example, we found the SLLP1-specific IgG antibodies to be restricted to a single peptide in the amino acid region 161–180 of the whole protein (Figure 3b). However, antibody responses detected in the blood and in the BM of our myeloma patients were mostly quite weak and sometimes barely exceeded the threshold defined by our analysis of sera from 112 healthy blood donors.

### SLLP1 is a potential immunogenic target for T cells

**Figure 4:**
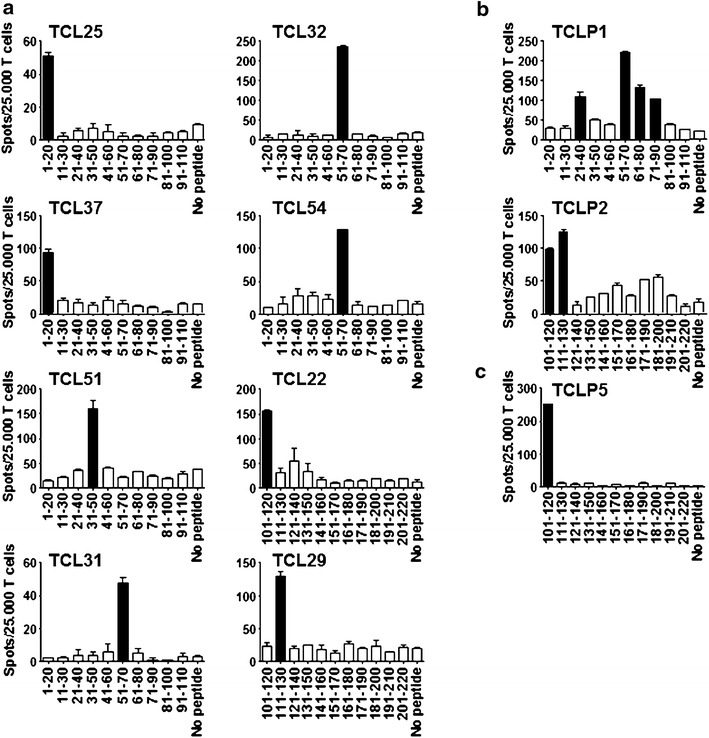
Induction of T cell specificity against multiple SLLP1 peptide epitopes. SLLP1-reactive T cell cultures were generated from PBMC from three healthy donors: **a** UKE-1, **b** UKE-2, and **c** UKE-3. Each plot represents an SLLP1-specific T cell line (TCL). IFN-γ secretion was measured in ELISPOT assay after restimulation with T-APC loaded with single peptides. T cells stimulated with unloaded T-APC were used as background controls (no peptide). *Bars* indicate the number of IFN-γ spots per 25,000 T cells (mean + SEM). *Black bars* indicate significant reactivity above the background value + 3STD.

**Figure 5:**
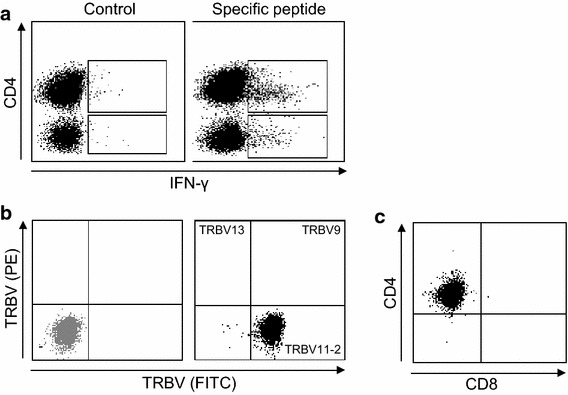
Phenotype of SLLP1-specific T cells. **a** T cell lines were restimulated with their cognate SLLP1_31–50_ peptide or irrelevant control peptide and secretion of IFN-γ was measured on the cell surface by flow cytometry. **b** T cell clone number 1 (TCC1) was stained with an antibody cocktail containing three different TRBV antibodies (PE, FITC, PE/FITC). The *gray dot plot* represents the isotype control. **c** SLLP-1-specific T cell clones analyzed phenotypically using staining with anti-CD4 and anti-CD8 antibodies followed by flow cytometry.

Since humoral and cellular immune responses against CTA often occur simultaneously [19], we selected 10 seropositive patients based on our ELISA results to analyze their peripheral blood for the presence of SLLP1-specific T cells. However, after one round of antigen-specific presensitization none of the investigated patients showed detectable levels of SLLP1-specific memory T cell responses (data not shown). Therefore, we decided to use a DC-based approach to investigate the capability of SLLP1 to induce de novo T cell responses. To this end, T cells from 3 healthy donors were repeatedly stimulated with autologous monocyte-derived DCs loaded with peptide pools containing 10 and 11 overlapping SLLP1 20-mer peptides, respectively. After 6–8 weeks of culture and repeated rounds of restimulation T cell lines were tested for the presence of SLLP1 reactivity. We detected anti-SLLP1 T cell responses among lines of all three donors tested (Figure 4a–c). These responses were directed against a variety of different SLLP1 epitopes with epitope SLLP1_101–120_ eliciting specific T cells in all donors tested (Figure 4a–c). When we analyzed the SLLP1-specific T cell lines generated by flow cytometry we were able to confirm that they specifically produced IFN-γ upon exposure to their cognate antigen (Figure 5a).

In a final step, we isolated a total of five different T cell clones (TCC) from the SLLP1-specific T cell lines generated. Clonality was confirmed by T cell receptor beta chain variable usage in all the TCC (Figure 5b). When we analyzed the phenotype of the TCC we found that all the clones were CD4+ (Figure 5c). Importantly, we were able to sequence the T cell receptors of all the 5 SLLP-specific TCC (Table 3) facilitating their use in future approaches targeting SLLP1 in myeloma, for example by the adoptive transfer of TCR-transduced T cells. Based on limited patient material we were not able to test tumor recognition by our SLLP1-specific T cell clones. However, studies evaluating safety and efficacy of the adoptive transfer of SLLP1 TCR-transduced T cells are currently ongoing in our lab.

**Table 3:** Sequencing of SLLP1-specific T cell receptors

	Peptide	Sequence	Purity (%)	TRBV	CDR3 Beta	TRAV	CDR3 Alpha
TCC 1	31–50	SCLSSQSSALSQSGGGSTSA	100	11-2	CASSLGAGGYTEAFF	8-3*01	CAVGGAGNMLTF
TCC 2	51–70	AGIEARSRALRRRWCPAGIM	98	10-3	CAISDGISGNTIYF	14	CAMRVGSDGQKLLF
TCC 3	1–20	MVSALRGAPLIRVHSSPVSS	–	–	–	–	–
TCC 4	111–130	SLADWVCLAYFTSGFNAAAL	99	12	CASSLTENIQYF	13-1	CAASSGGSNYKLTF
TCC 5	51–70	AGIEARSRALRRRWCPAGIM	–	12-4	CASSFGQPNLNYGYTF	19	CALSEAGFQKLVF

Naming of TCR chains according to IMGT nomenclature. SLLP1 peptide and CDR3 sequence are displayed using amino acid single letter code. T cell clone purity was determined by flow cytometry.

*TRBV/TRAV* T cell receptor variable beta/alpha chain, *CDR3* complementarity determining region 3.

## Discussion

We have here analyzed the value of a potential new target structure, cancer-testis antigen SLLP1, for the treatment of MM. One major criterion for the applicability of a given tumor antigen for cancer immunotherapy is that it shows a tumor-restricted expression and is absent from the essential types of healthy tissues. Condomines and coworkers previously observed RNA expression of SLLP1 in a proportion of normal peripheral blood B cells and in polyclonal plasmablasts generated in vitro from normal B cells but not in BM plasma cells from healthy donors [20]. In contrast, Wang et al. did not detect any SLLP1 expression in peripheral blood and BM samples from healthy donors [11]. We were able to confirm our previous finding that SLLP1 is absent from whole BM cells from healthy donors [21] in our current investigation. All available evidence, therefore, supports the idea that SLLP1 is not present in normal hematopoietic cells and in that sense shows a tumor-restricted expression. In addition, publicly available databases indicate an absence of SLLP1 from most normal epithelial tissues [22]. However, there is one notable exception with significant RNA-level expression in the pancreas. While this does not necessarily translate to protein-level expression, future studies should perform a careful assessment of SLLP1 expression in such normal tissues before considering SLLP1 as a therapeutic target in MM.

Another important point is that the tumor antigen in question should show a sufficiently frequent tumor-related expression to enable its use in a substantial proportion of patients with a given malignancy. We observed that the vast majority of myeloma cell lines expressed SLLP1 on the RNA and protein levels. In addition, we detected SLLP1 expression in the PC-containing BM of 44% of patients with newly diagnosed MM. These findings are in accordance with previous results on SLLP1 expression in myeloma. SLLP1 has been found to be expressed in hematologic malignancies [11, 21, 23] including MM [11, 20]. In an initial study, 6 out of 17 myeloma patients analyzed were found to evidence SLLP1 RNA expression [11]. Another study observed an expression of SLLP1 in 33% of samples from 64 myeloma patients using microarray technology [20]. Overall, the combined data suggest that SLLP1 is among the CTA most frequently expressed in MM.

A given tumor antigen is probably better suited as a therapeutic target if it has a central function in the biology the respective cancer type because this would keep the malignancy from downregulating its expression under the selective pressure of an immunotherapeutic approach. The biological function of SLLP1 in human cancers has never been investigated but our current results indicate that this CTA might very well play a role in the development and/or progression of MM as we and others have demonstrated for other CTAs frequently expressed in MM [24, 25]. We found, for example, SLLP1 expression in all our patient-derived whole BM samples to correlate with a number of negative prognostic factors including the patient’s age, the number of BM-infiltrating plasma cells, as well as serum levels of albumin, β_2_-microglobulin, hemoglobin, and creatinine. In addition, we observed an extraordinarily frequent expression of SLLP1 in patients with adverse cytogenetics, in particular patients who evidenced a t(4;14). One finding that also pointed to a role of SLLP1 in the pathophysiology of MM is our observation that SLLP1 expression was to a certain degree associated with an adverse prognosis in patients treated with alloSCT who had evidenced SLLP1 expression in their BM at least at one time point after transplant. It is not entirely clear why this association was only present in the group of patients who had received alloSCT as part of their overall therapeutic concept, however, it could simply be related to the fact that these patients represented the largest group included in our study. On the other hand, SLLP1 could potentially represent a target for an immunologic graft-versus-myeloma effect [26–28] and we cannot exclude the possibility that our observation of a significantly lower expression of SLLP1 after alloSCT is based on a transplant-induced eradication of SLLP1-expressing tumor cells from the patients’ BM.

It might be of benefit for the therapeutic value of a certain antigen if it is capable of spontaneously inducing immune responses because immunotherapeutic interventions could potentially build on such a preexisting immunity. Our previous investigations suggested that antibody responses are commonly associated with T cell responses against the same antigen [19, 29, 30]. Therefore, we decided to first screen our large collection of patient samples for humoral responses against SLLP1 and then focus on the SLLP1-antibody-positive patients and analyze them for the presence SLLP1-specific T cells. We found that about 9.5% of our patients evidenced antibody responses against SLLP1. The prevalence of humoral responses against SLLP1 would, therefore, be significantly higher than the frequency of antibody responses against two other CTA, namely NY-ESO-1 and SSX-2, we had observed in one of our very recent studies [30]. However, in contrast to the latter two CTA, antibody responses against SLLP1 were mostly low-titered. Consequently, a comparably diminished uptake of SLLP1 immune complexes by professional APC and the resulting compromised presentation of tumor antigen to T cells may also represent one underlying reason for our finding that none of the seropositive patients evidenced spontaneous T cell responses against SLLP1 [31–33].

We did not detect any surface expression of SLLP1 on myeloma cells by flow cytometry and CTA are generally known for their intracellular protein expression [34]. As a consequence, these tumor antigens cannot be targeted by therapeutic monoclonal antibodies or chimeric antigen receptor (CAR)-transduced cells. Classical T cell-based approaches such as tumor vaccination seem more appropriate, however, two large phase III vaccination studies using recombinant proteins of CTA MAGE-A3 for lung cancer and melanoma, the MAGRIT and DERMA trials, have recently failed. We, therefore, believe that alternative modes of treatment, such as the adoptive transfer of T cell receptor (TCR)-transduced T cells are probably more likely to show clinical efficacy in the immunotherapy of cancer using CTA as targets. Accordingly, clinical studies using CTA NY-ESO-1-specific TCR-transduced T cells for the treatment of patients with melanoma and synovial cell sarcoma have shown remarkable response rates [35, 36]. On the other hand, one study applying T cells transduced with a TCR specific for CTA MAGE-A3 was associated with severe toxicity based on the fact that a homologue to MAGE-A3, namely MAGE-A12, was expressed in the patients’ brain tissue [37]. It might, therefore, be of advantage that SLLP1 does not show any homology to such antigens known for their unspecific expression in healthy tissues.

In conclusion, we have demonstrated here that cancer-testis antigen SLLP1 shows a tumor-restricted expression pattern and is present in the tumor cells of a substantial proportion of patients with MM. The expression of SLLP1 is associated with a number of adverse clinicopathological parameters and a worse outcome of myeloma patients. Spontaneous anti-SLLP1 humoral immunity occurs comparably frequently in MM but antibody responses are mostly low-titered and spontaneous T cell responses were absent. However, de novo T cell responses against SLLP1, which can be exploited for adoptive cell transfer approaches, can easily be induced. Thus, SLLP1 represents a promising target for the immunotherapy of MM and, hopefully, our combined findings will lead to the development of effective immunotherapeutic approaches for patients with this fatal hematologic malignancy.

## Conclusions

Most patients with MM will relapse after an initially successful therapy and eventually succumb to their disease. Immunotherapeutic approaches could contribute to eradicating chemotherapy-resistant tumor cells from the patients’ BM and facilitate prolonged remissions or even cures. Evaluating cancer-testis antigen SLLP1 as a potential immunotherapeutic target for MM we found myeloma-specific expression of the target antigen in the BM of a substantial proportion of myeloma patients. Expression of SLLP1 was associated with adverse cytogenetics and with a number of other negative prognostic factors. Among patients treated with allogeneic stem cell transplantation those with SLLP1 expression showed a reduced overall survival. Finally, we detected spontaneous anti-SLLP humoral immunity in myeloma patients and SLLP1-specific T cells could readily be induced in vitro after stimulation with SLLP1. Our combined observations suggest that cancer-testis antigen SLLP1 represents a promising target for the immunotherapy of MM, in particular for the adoptive transfer of T cell receptor-transduced T cells.
